# The impact of healthcare provision on immigrant pregnancy behaviors: the case of Ramadan fasting in Germany

**DOI:** 10.1016/j.jmh.2025.100349

**Published:** 2025-07-25

**Authors:** Paul Witte, Fabienne Pradella, Reyn van Ewijk

**Affiliations:** a Johannes Gutenberg University Mainz, Department of Business and Economics, Chair of Statistics and Econometrics, Jakob-Welder-Weg 4, Mainz, Germany; b Heidelberg Medical Faculty and University Hospital, Heidelberg Institute of Global Health, Im Neuenheimer Feld 130.3, Heidelberg, Germany; c Stanford University, Division of Primary Care and Population Health, 291 Campus Drive, Stanford, USA

**Keywords:** Prenatal care, Ramadan, Pregnancy

## Abstract

Germany and other Western countries are home to a growing number of Muslims. This implies that health-related behaviors more common among Muslims are becoming increasingly important in routine healthcare. For example, Ramadan during pregnancy has been shown to be associated with adverse offspring health outcomes along the life course. At the same time, a high share of pregnant Muslims worldwide chooses to fast. In this study, we investigate the dynamics underlying Ramadan fasting during pregnancy in Germany, using survey data (N=326) of Muslims delivering after being pregnant during a Ramadan. In this sample, 36.5 % of women fasted during their recent pregnancy, for an average of 17 days. Respondents generally did not regard fasting during pregnancy as obligatory and women tended to make their own, independent decisions about whether to fast. Most women did not expect fasting to be associated with impaired offspring health, even though many women actively searched for information on Ramadan during pregnancy. They often did so on the internet or by talking to family and friends. Only about one-third of women consulted with their prenatal caregiver about fasting. These consultations were associated with a reduction in days fasted by about 11 days. A sensitization of healthcare providers to Ramadan during pregnancy, and routinely addressing the issue with Muslim families of reproductive age can have important public health benefits.

## 1 Introduction

Many countries in the Global North are home to growing Muslim populations. In Germany, Muslims currently constitute 6.4 to 6.7 % of the general population (Pfündel et al., 2021). Historically, starting in the 1960s, the Federal Republic of Germany recruited foreign guestworkers from Mediterranean countries to provide additional labor for the rapidly growing post-war domestic industries. Among those were many Muslims, mostly from Turkey, but also from countries such as Tunisia or Morocco (Kastoryano, 2004). More recently, the number of immigrants with a Muslim background began to increase in the early 2010s, with Syria currently being the most common origin country (Türkmen, 2024).

While it is important to provide access to healthcare for all, navigating the healthcare system can be a challenge for individuals with a migration background, including Muslims (Lebano et al., 2020, Aljadeeah et al., 2021). This is particularly the case for prenatal care. Beside the potential issue of language barriers, people who have grown up in a different country may be less familiar with their new home country’s healthcare system or may have different perceptions about pregnancy and health (Alomair et al., 2020). Healthcare providers, in turn, may address health-related behaviors that are more specific to populations with a migration background less frequently or less adequately due to unfamiliarity. One example for this is the observance of Ramadan.

In the Muslim holy month of Ramadan, healthy adult Muslims engage in 29-30 days-long intermittent fast wherein they abstain from food and drinks during daytime. While pregnant women are exempted from the fast according to most Islamic interpretations, evidence from around the world shows that a substantial share of Muslim women does engage in Ramadan fasting while pregnant. This applies to both Muslim-majority countries and the growing Muslim minorities in traditionally non-Muslim countries, with fasting rates among pregnant women ranging from 43 % in the UK, 54 % in the Netherlands, 88 % in Pakistan to 99 % in Bangladesh (Mubeen et al., 2012, Petherick et al., 2014, Savitri et al., 2014, Seiermann et al., 2021). At the same time, Ramadan during pregnancy has been shown to be associated with a range of adverse health outcomes among the offspring. These range from poorer cognitive scores and increased mortality rates in childhood, to increased rates of disabilities and a higher risk of chronic diseases in adulthood (Pradella et al., 2024).

For expectant Muslim women, adherence to Ramadan can be regarded as one of various behavioral decisions made during pregnancy. A peculiarity of Ramadan fasting decisions is that next to health concerns, they are subject to religious and cultural customs, norms, and beliefs. Health-related behavior is the result of a decision-making process with various internal and external input factors (McEachan et al., 2016, Hagger et al., 2018, Conner et al., 2017). Personal beliefs, attitudes, capabilities, and beliefs about how one’s environment perceives the enacted behavior determine behavioral intentions. Moderated by social and socio-economic environmental constraints, these intentions translate into actual behavior (Fishbein and Ajzen, 2011).

In this study, we use survey data containing detailed individual-level information on pregnant Muslims’ fasting behavior in a recent Ramadan, their encounters with prenatal care regarding Ramadan fasting during pregnancy as well as a rich set of socio-demographic characteristics. After providing detailed evidence on the intentions, reasons, information sources and social characteristics of pregnant Muslims who decide (not) to fast during Ramadan, we empirically investigate the role of healthcare in the complex web of social norms that shape behavior during pregnancy.

Healthcare can be considered as an external influencing factor for the maternal fasting decision, and this study is the first to empirically investigate the relationship between prenatal consultations and maternal Ramadan fasting during pregnancy.

We aim to contribute to a better understanding of how healthcare providers in Western countries can improve support to Muslim patients. In high-income countries, routine prenatal care reaches almost all pregnant women, and assists them in navigating safely through their pregnancy (Shaw et al., 2016). A sensitization of prenatal healthcare providers to behaviors during pregnancy that are more common among specific immigrant populations can contribute to a healthy start to life for all children, as envisioned by the Sustainable Development Goal 3.

## 2 Background

### 2.1 Prenatal care and maternal diet during pregnancy in Germany

In Germany, routine prenatal care covered by statutory health insurance is provided by gynecologists, obstetricians, and midwives. Maternity care guidelines and regulations are issued and updated regularly by the Federal Joint Committee of the German healthcare system (G-BA) (Gemeinsamer Bundesausschuss 2023). During routine monthly visits (bi-weekly past the 32^nd^ gestational week), the caregiver is mostly concerned with detecting potential complications or anomalies and mitigating or avoiding maternal or fetal (neonatal) morbidity and mortality (Tempfer, 2014), via various screening methods.

During the first routine visit, the medical history of the patient, as well as her current mental and social situation following a list of 26 items outlined in the official maternity records (“Mutterpass”) as issued by the G-BA, are documented. Furthermore, consultation is provided on nutrition, medication, alcohol, tobacco, drugs, work, sport and traveling during pregnancy. The guidelines are rather unspecific regarding the content of the consultation on diet and nutrition during pregnancy. The only explicit recommendations are to increase maternal iodine supplementation by 100 to 200 µg per day and to ensure stable glucose levels among patients with a risk for gestational diabetes. Aside from that, the prenatal caregiver is to provide some general consultation on nutrition, but this is not specified any further.

Recommendations issued by other German scientific organizations and networks more explicitly address diet and nutrition. A set of “National Consensus Recommendations” emphasizes increased requirements for certain nutrients, vitamins, and trace elements during pregnancy (Koletzko et al., 2013). The German Society for Nutrition (DGE 2024) issued uniform recommendations for diet and nutrition during pregnancy that are mostly congruent with the ones published by Koletzko, et al. (Koletzko et al., 2013). Other studies published in the German language area additionally highlight the importance of a balanced diet (Kleine-Gunk, 2021, Zauner, 2022). These recommendations include a stable caloric intake with regular meals, high quality and balanced diets rich in essential nutrients, and hydration. Intermittent fasting during pregnancy is not addressed directly in the maternity guidelines discussed above.

### 2.2 Nutrition and intermittent fasting during pregnancy and offspring health

During Ramadan, Muslims engage in customs and traditions, one of which is intermittent (daytime) fasting which includes abstinence from both food and drink. Ramadan may be beneficial to metabolic and cardiovascular health in healthy adults (Al-Jafar et al., 2021, Mirmiran et al., 2019, Adawi et al., 2017, Madkour et al., 2023) and at the same time improve life satisfaction and prosocial behavior (Campante and Yanagizawa-Drott, 2015, Haruvy et al., 2018). Pregnant women, however, have a relatively low tolerance threshold for nutritional restrictions due to their already elevated energy demands (Hobel and Culhane, 2003). Because of this, intermittent fasting during pregnancy can lead to metabolic and hormonal signs comparable to those observed during starvation (Metzger et al., 1982).

Most Muslim pregnancies overlap with a Ramadan and many pregnant Muslim women decide to fast. In most countries, a majority of pregnant Muslims fast during Ramadan, with rates ranging from 43 % in the UK, 54 % in the Netherlands, 88 % in Pakistan to 99 % in Bangladesh (Mubeen et al., 2012, Petherick et al., 2014, Savitri et al., 2014, Seiermann et al., 2021).In Germany, around 40 % of pregnant Muslims reported fasting during Ramadan (Pradella et al., 2023).

Prenatal exposure to a suboptimal nutrition can result in a re-allocation of scarce resources to the development of the most vital organs (for example brain and heart) at the expense of other, less vital organs and tissues (for example kidneys). While these mechanisms have advantages from an evolutionary perspective - ensuring survival - these adaptations have been shown to come at the cost of an earlier onset of chronic diseases in adulthood (Godfrey and Barker, 2001). Exposure during early pregnancy is particularly harmful, when DNA methylation is highly sensitive (Harris and Seckl, 2011, Griffiths and Hunter, 2014, Tobi et al., 2015).

Many of these health effects may be latent and thus not visible during pregnancy or around the time of birth and only manifest in adulthood. This is also reflected in the literature on Ramadan during pregnancy (Pradella et al., 2024). Even though effects on measurable birth outcomes are small or absent, various long- and medium-term consequences have been documented. This includes symptoms indicative of type II diabetes and coronary heart disease among the offspring (Van Ewijk, Dec 2011), lower height-for-age and smaller stature (Chaudhry and Mir, Apr 28 2021, Karimi and Basu, 2018, Kunto and Mandemakers, 2019, Van Ewijk et al., Apr 15 2013), an increased mortality among under 5 year-olds (Schoeps et al., 2018) and increased risks for respiratory symptoms (Pradella and van Ewijk, 2018), as well as reduced success at school and on the labor market (Almond et al., 2015, Majid et al., 2019, Majid, 2015, Almond and Mazumder, 2011).

### 2.3 Decision-making underlying Ramadan fasting during pregnancy

Ramadan fasting is deeply embedded into Muslim culture and traditions. Starting in adolescence, Muslims are being accustomed to engaging in daytime fasting during the month of Ramadan. Breaking the fast with traditional meals together with family and friends may provide a pleasant and communal experience that an individual would prefer to not miss out on. Furthermore, the month of Ramadan is regarded as a time of spirituality and personal reflection. For most healthy adult Muslims, fasting during Ramadan is simply the normal thing to do (Goitein, 2017).

It is important to note that for pregnant women, different rules on fasting during Ramadan apply than for other adult Muslims, and they are exempt from the fasting obligation if they fear health consequences from fasting for either themselves or the unborn child (Abolaban and Al Moujahed, 2017). Pregnant women might therefore contemplate their fasting decisions differently, conditional on being aware of the pregnancy. Since the knowledge of adverse health effects may be sufficient for the woman to at least reconsider her fasting intentions, updating the woman’s information stock on potential health risks for the baby could influence her decision-making. This requires that a prenatal caregiver can at least exert some influence on the maternal fasting decision.

The decision-making process underlying health-related behaviors is described by psychological theory. The individual’s attitudes and subjective norms determine behavioral intentions (McEachan et al., 2016). Here, attitudes include the woman’s (religious) beliefs about fasting being generally good, and whether the (health) outcome of fasting is evaluated to be good. A caregiver could directly influence this by informing about potential health risks for the offspring. Subjective norms describe the individual’s beliefs about how the enacted behavior is perceived by the relevant social environment, which in this context may include the partner, family, or community. In other words, a woman’s perception of what the immediate social environment may think of her fasting directly influences her intentions. If considered to be part of the relevant social environment, the perceived attitude of the caregiver toward fasting during pregnancy may become part of the subjective norms and therefore affect the woman’s intentions as well.

Depending on environmental constraints and behavioral control, intentions then translate into actual behavior. Difficulties to deviate from everybody else’s eating and drinking rhythms or not wanting to reveal an early pregnancy by not fasting might be such constraining factors. Overall, this suggests that in principle, a prenatal caregiver should be able to exert some influence on maternal Ramadan fasting behavior during pregnancy. Prenatal care provision has been shown to be influential on other maternal health behaviors: Terza, et al. (Terza et al., 2008) showed empirically that prenatal consultations reduced maternal alcohol consumption, while Yan (Yan, 2017) showed that insufficient prenatal care utilization increased the risks of maternal smoking during pregnancy, and postpartum smoking.

The decision-making processes underlying Ramadan fasting during pregnancy have remained largely unexplored, including the role of prenatal care. This study first describes the reasons, motivations, and expectations about maternal Ramadan fasting during pregnancy among pregnant Muslims in Germany. Previous research suggest that Muslim women appreciate healthcare provision being respectful of and informed about customs and traditions such as Ramadan (Lou and Hammoud, 2016). We therefore focus on the role of prenatal consultations by assessing whether their provision is associated with changes in maternal fasting behavior.

## 3 Methods

### 3.1 Data

We use data from a survey conducted in 2017 and 2018 among pregnant Muslims in Mainz, Germany. Study participants were interviewed in the obstetric wards of the two hospitals in the city shortly before or after giving birth. The survey captures an entire cohort of pregnant Muslims, so that we have data on the overlap of pregnancy and Ramadan in all pregnancy trimesters. The interviews were conducted in German, English, Turkish and Arabic. The target group consisted of those women who delivered in Mainz and whose current/recent pregnancy overlapped with the month of Ramadan and who spoke one of the four interview languages (Turkish, Arabic, German, English).

**Fig. 1:**
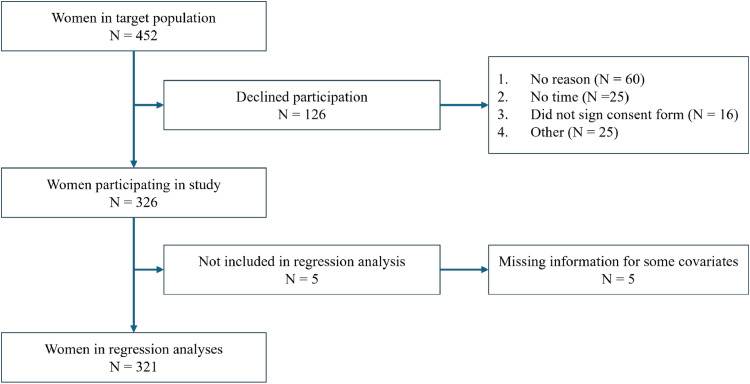
Sample selection from the Mainz survey study on Ramadan during pregnancy.

Of the 452 women considered to be part of the target group, 326 were willing to participate in the survey, yielding a response rate of 72 %. This high response rate ensures that we capture a high and relevant share of the target population. Five observations could not be considered in the final regression analysis due to missing values (see Figure 1). The survey is described in more detail in Pradella et al. (2023).

### 3.2 Descriptive analysis

We describe the socio-demographic characteristics of our sample to provide relevant insights into the composition of the Muslim population in Germany that does engage in Ramadan fasting during pregnancy. Moreover, we compare the characteristics of women based on their fasting status (whether the woman did engage in Ramadan fasting during pregnancy or not), as well as their prenatal consultation status (whether the woman did receive prenatal consultation on Ramadan fasting or not). Both stratifications provide information as to whether women who fast during pregnancy or who receive prenatal consultation on the matter differ substantially from those who do not. In doing so, this study provides a first understanding of the Ramadan fasting population and its interaction with the routine prenatal care system in Germany. Furthermore, we describe motivations for or against fasting, as well as information sources on the topic that had been consulted prior to the fasting decision.

### 3.3 Outcome variables of regression analysis

We also empirically estimate associations between prenatal consultation on Ramadan during pregnancy and fasting during pregnancy. The outcome of interest, maternal fasting during pregnancy, is captured by two variables. Participants were asked whether they had not fasted, fasted a few days (1-2 days), some days (3-9 days), around half of Ramadan (10-19 days) or most or all days (20-29 days). 58.8 % of fasting women provided an exact number of fasted days. For the remaining women, the number of fasted days was computed as the midpoint of the indicated fasting intensity category (see above). Based on the latter two measurements, we compute a continuous measure of the number of fasted days by combining the exact measures and the midpoints from the categorical statements into a single composite measure.

### 3.4 Explanatory variable of regression analysis

Having received prenatal consultation on Ramadan fasting during pregnancy is the explanatory variable of interest. We construct a binary variable based on a survey item that asked survey participants to indicate whether they had received consultation on Ramadan fasting by a prenatal caregiver. Additionally, the content of the advice provided by the caregiver was recorded in an open-ended question. None of the advice that was provided during consultation in our sample was encouraging of Ramadan fasting during pregnancy.

### 3.5 Regression analysis model and covariates

Because pregnant Muslims make two choices regarding Ramadan fasting during pregnancy – first, whether they fast at all, and second – if they decide to fast – for how many days, we use a log-normal hurdle (LH) model (Cragg, 1971). The LH Model accounts for these two choices by estimating the effects on the extensive margin (the probability to participate in fasting) and the intensive margin (the number of fasted days conditional on participation) separately. The regression model is detailed in the online appendix A.1.

It is pivotal to incorporate a set of covariates in these analyses, since individual characteristics could both influence the decision (not) to fast and the decision to discuss Ramadan fasting with a prenatal caregiver. We control for the woman’s age, country of birth indicators and the pregnancy trimester during which the woman’s pregnancy overlapped with Ramadan. Furthermore, we adjust for employment status and educational attainment (both included as categorical variables indicating employment type and highest educational degree respectively). We also control for the degree of religiosity by including a variable indicating whether the woman wears a veil on a daily basis. Routinely wearing a veil can be considered a proxy for religiousness (Brünig and Fleischmann, 2015). Evidence from Robinson and Raisler (Robinson and Raisler, 2005) suggests that religious women might be more inclined to participate in Ramadan fasting and less inclined to discuss this topic with a healthcare provider. If Ramadan is not observed by a woman’s immediate environment, she might be less inclined to fast during her pregnancy. We control for this by including a variable indicating whether any household member of the woman adhered to Ramadan fasting. Similarly, the opinions and attitudes of the woman’s partner may have a formative effect on fasting choice and responsiveness to medical consultation. Thus, we control for the partner’s objection, measured by a binary variable indicating whether the partner was opposed to Ramadan fasting during pregnancy. In two robustness checks, we additionally controlled for whether the woman had fasted in a previous pregnancy and whether she had informed herself about Ramadan fasting during pregnancy via other sources (e.g. media). We did not include these covariates in the main specification because only a subset of women reported on fasting during previous pregnancies resulting in a substantial sample size reduction and information seeking is potentially endogenous (as it may be the result of the consultation).

A potential concern in our analyses is that the set of covariates may not sufficiently deal with the fact that prenatal consultation on Ramadan during pregnancy was not randomly assigned. To the extent that the consultation on Ramadan fasting was initiated by the woman and not the caregiver (i.e. women asking about Ramadan during pregnancy during a routine check-up), it is possible that women self-select into prenatal consultation not only based on their observed characteristics – for which we control -, but also on unobserved characteristics. As a sensitivity analysis, we therefore employ the Oster method to test how likely it is that results are driven by residual confounding (Oster, 2019).

The idea of the Oster method is to test how stable an estimated effect (in our case of prenatal consultation on the fasting behavior) is by including additional covariates (here the socio-demographic control variables listed above). Adding covariates should reduce bias. Ideally, one should have all relevant covariates in one’s database, so that all bias is taken out. However, in practice, only a limited set of covariates is available. By assessing whether the regression coefficient of interest changes considerably when covariates are added, one can gauge whether it would still change more when all relevant covariates (including the “unobservable covariates” not included in the dataset) would have been included. If the coefficient of interest is very sensitive to the adding of covariates, then there is a high likelihood that it will be subject to residual bias. The Oster method formalizes this by estimating how relevant unobservable covariates would need to be to explain away the estimated association between prenatal consultation on fasting behavior. For more details, see online appendix A.2.

All analyses were conducted using STATA SE version 17.

## 4 Results

### 4.1 Descriptive evidence

Table 1 shows sample characteristics for the full sample, as well as stratified by whether the respective woman did engage in Ramadan fasting during pregnancy, and by whether the woman received prenatal consultation on the matter. Among all survey participants, 36.5 % indicated that they had fasted at least one day during Ramadan, and 33.5 % had consulted with their prenatal care provider about fasting during pregnancy. The majority of women (72.1 %) were first generation immigrants, with roughly equal shares coming from Turkey, Syria, Morocco and other Arab countries. Note that all German-born women in our sample indicated to be second-generation immigrants. More than half of the women wore veiling daily, and about 85 % of women were in households in which members fasted. Moreover, 63.5 % of partners objected to fasting during pregnancy. This share was higher among women who did not fast, but even among women who did choose to fast, 45.4 % reported having a partner who objects to doing so.

**Table 1:** Descriptive statistics stratified by Ramadan fasting and prenatal consultation reception.

	Full sample	Ramadan fasting			Prenatal consultation		
	(N=326)	Yes (N=119)	No (N=207)	P-value difference	Yes (N=109)	No (N=216)	P-value difference
Fasting share	36.5	100.0	0.0	(n.a.)	50.5	29.6	<0.01^b^
Days fasted	6.2	17.0	0.0	(n.a.)	7.7	5.5	0.08^a^
Prenatal consultation share	33.5	46.2	26.2	<0.01^b^	100.0	0.0	(n.a.)
**Education**				0.20^c^			0.63^c^
Low	13.8	18.5	11.1		16.5	12.5	
Medium	55.8	52.9	57.5		48.6	59.3	
High	30.4	28.6	31.4		34.9	28.2	
**Employment**				<0.01^b^			0.13^b^
Full-time employed	21,8	10.9	28.0		14.7	25.0	
Part-time	17.2	15.1	18.4		15.6	18.1	
Homemaker	37.4	47.1	31.9		40.4	36.1	
Student	15.0	22.7	11.1		20.2	12.5	
Unemployed	8.6	5.0	10.6		9.2	8.3	
**Country of birth**				<0.01^b^			<0.01^b^
Germany	27.9	12.6	36.7		22.9	30.6	
Syria	15.9	26.9	9.7		15.6	16.2	
Turkey	11.9	6.7	14.9		10.1	12.5	
Morocco	14.7	26.9	7.7		25.7	9.3	
Other Arab country	14.1	13.5	14.5		14.7	13.9	
South/Central Asia	8.3	5.9	8.7		6.4	9.3	
Other country	7.1	6.7	7.3		4.6	8.3	
**Pregnancy, religion and household related**							
First generation immigrant	72.1	87.4	63.3	<0.01^b^	77.1	69.4	0.15^b^
Less than 3 years living in Germany	27.6	42.0	19.3	<0.01^b^^a^	30.3	26.4	0.46^b^
Other household members fast	84.8	97.5	77.5	<0.01^b^	93.6	80.8	<0.01^b^
Partner objects to fasting during pregnancy	63.5	45.4	73.9	<0.01^b^	55.1	67.6	0.03^b^
Daily veiling	57.7	80.7	44.4	<0.01^b^	77.9	47.7	<0.01^b^
Woman’s age	30.1	29.9	30.3	0.59^a^	30.4	29.9	0.48^a^
Nulliparous	35.9	35.3	36.2	0.87^b^	29.4	39.4	0.08^b^
**Trimester overlapping with Ramadan**				<0.01^b^			0.19^b^
Trimester 1	36.0	47.9	29.1		30.3	39.1	
Trimester 2	28.3	24.4	30.6		33.9	25.6	
Trimester 3	35.7	27.7	40.3		35.8	35.4	

*Notes:* Values for categorical variables are reported as column percentages, for continuous variables (woman’s age, years living in Germany) mean values are reported. Low education is no degree/ elementary school; medium education is secondary school or vocational training; high education is technical or university degree.

a Independent samples *t-*test

b $\chi^{2}$-test

c Mann-Whitney U test

The stratification based on the fasting status furthermore reveals differences in employment types between the two groups, with a larger share of homemakers in the fasting group and a higher share of full-time employed women in the non-fasting group. The two groups also differed in their origin countries, with Syria and Morocco being the most frequent countries of birth in the fasting group and Germany in the non-fasting group (online appendix A.3). Fasting women also reported more often that other members of their household fasted and that they veiled on a daily basis. There was no significant difference in education levels, age, or parity. Fasting was more common among mothers who experienced Ramadan during their first pregnancy trimester.

The right part of the table compares survey participants who had vs had not received prenatal consultation on Ramadan fasting. Women who received prenatal consultation on Ramadan were more likely to fast during pregnancy (50.5 % vs 29.6 %). There is likely a component of self-selection in this as those women who deliberate on fasting are more likely to discuss it with a caregiver. Women who received a prenatal consultation on Ramadan were furthermore more likely to wear a veil on a daily basis and to live in a household in which other members adhered to the Ramadan fast. Furthermore, their partner was less likely to object to Ramadan fasting during pregnancy. Receiving prenatal consultations on Ramadan fasting did not differ significantly between first-generation immigrants and German natives or by whether women lived in Germany for less than three years. Information on reception of prenatal consultation was missing for one individual in our sample.

In addition, survey participants were asked to indicate their individual motivation for or against Ramadan fasting during pregnancy. The non-exclusive responses and their respective frequencies are displayed in Table 2. While many fasting women mentioned that they fasted because of religious reasons, this does not imply that they believed fasting was mandatory. Thirteen women (10.9 %) mentioned that fasting is obligatory, but five of these explicitly added to their reasoning that they were doing well, suggesting that they did not perceive fasting to be mandatory regardless of any health effects. It is possible that this reasoning applied to some of the other eight women as well. Overall, the most-mentioned reason for fasting was that women wanted to try fasting while being pregnant and made a positive experience with this. No woman said that someone else determined for her that she should fast.

**Table 2:** Individual motivations for or against Ramadan fasting during pregnancy.

	Motivation	
	N	Share (Percent)
*Fasting women (N = 119)*		
Wanted to try, and it went well	30	25.2
Did not know yet that she was pregnant	20	16.8
Fasting is mandatory according to religion	8	6.7
Fasting is mandatory and it went well	5	4.2
Is used to the fasting custom	4	3.4
Having to make up for the missed fast afterwards	3	2.5
Religion (general, not further specified)	43	36.1
*Non-fasting women (N = 207)*		
Diagnosed health condition (pre-existing conditions, (pregnancy) diabetes, medication usage, etc.)	42	20.3
Nausea, tiredness, circulatory problems	33	15.9
Concerns for health of child	18	8.7
Fasting not mandatory during pregnancy according to Islam	16	7.7
Never fasts during Ramadan	12	5.8
This year, Ramadan was in summer (long/hot days)	10	4.8
Work	2	1.0
Husband did not consent to fasting	1	0.5
Pregnancy (general, not further specified)	87	42.0

Note: Women could state more than one reason for/against fasting

Among the women who did not fast, a large share (42.0 %) cited pregnancy in general, which may encompass concerns about their own health or that of their baby, as a reason why they decided against fasting. Furthermore, participants reported diagnosed health conditions, either pre-existing or pregnancy-related (20.3 %), and cited symptoms such as nausea, fatigue, or circulatory problems (15.9 %) as reasons for not fasting. A share of 8.7 % of non-fasting women explicitly mentioned concerns about the health of the child. At the same time, when asked about expectations for child health, more than 50 % of non-fasting women stated that they expect fasting to be associated with adverse effects for their offspring (Table 3). Few women (5.8 %) reported never to be fasting during a Ramadan. Again, husbands and other family members were rarely mentioned.

**Table 3:** Differences in health effect expectations between fasters and non-fasters (column percentages).

Expected effect on child’s health	Not fasting		Fasting		
	N	Share (Percent)	N	Share (Percent)	
No effect	36	17.4	54	45.4	90
Negative effect	116	56.0	23	19.3	139
Positive effect	15	7.3	21	17.7	36
Depends on mother’s health	30	14.5	15	12.6	45
Other (don’t know / depends on pregnancy phase or situation)	10	4.8	6	5.0	16
Total	207	100.0	119	100.0	326
Comparison of fasting vs non-fasting women: $\chi^{2}$= 52.9 (p-value < 0.01)					

Expected effect on mother’s health	Not fasting		Fasting		
No effect	32	15.5	52	43.7	84
Negative effect	122	58.9	29	24.4	151
Positive effect	23	11.1	13	10.9	36
Depends on mother’s health	15	7.3	14	11.8	29
Other (don’t know / depends on pregnancy phase or situation)	15	7.3	11	9.2	26
Total	207 100.0		119 100.0		326

Comparison of fasting vs non-fasting women: $\chi^{2}$= 45.0 (p-value < 0.01)

Table 3 gives more detail on the health effects that fasting and non-fasting women expected. A substantial share of women did not expect adverse effects of fasting on the health of the child. Among fasting women, over 63 % expected no or positive health effects on the child. 19.3 % of the fasting women fasted despite believing that this could have negative health effects on the child. A further 12.6 % believed this to depend on the mother’s health. Among non-fasting women, 56.0 % expected negative effects on the health of the child, and a further 14.5 % thought this to depend on the health of the mother. A slightly higher share of both fasting and non-fasting women expected negative health effects on the mother rather than the child.

As shown in Table 4, 45 % of the women actively sought information about Ramadan during pregnancy. This share was significantly higher among fasting women. The internet was consulted most frequently, followed by family and friends. Religious experts and the Quran were rarely mentioned as sources of information. Furthermore, to better understand the information-seeking behavior of fasting women, we compared the number of fasted days and health-related beliefs by information source used (table A.4 in the online appendix). We compared four groups: women who consulted only a medical professional, those who sought information exclusively from other sources (e.g. media), those who used both sources, and those who did not seek information at all. Across all outcome domains, we found no statistically significant differences between these groups.

**Table 4:** Information sources consulted prior to Ramadan.

	(1) Full sample (Percent)	(2) Fasting (Percent)	(3) Not fasting (Percent)	(4) P-value (difference between (2) and (3))
Any information	45.1	57.1	38.2	< 0.01
Internet sources	31.7	40.9	26.0	< 0.01
Family	21.3	25.9	18.5	0.14
Friends	9.0	9.5	8.7	0.81
Books or magazines	6.9	5.8	7.5	0.58
Religious expert	4.9	6.7	3.9	0.25
Quran	1.5	0.8	1.9	0.66

Note: P-values in column (4) based on $\chi^{2}$-test and (for small cells size) Fisher’s exact test

### 4.2 Associations between prenatal consultation on Ramadan and fasting behavior

The results of our regression analysis on the associations between prenatal consultations on Ramadan during pregnancy and maternal fasting behavior are summarized in Table 5. It presents the results from our log-normal hurdle regressions. Results are shown as average partial effects, meaning that the coefficients in panel A reflect the change in the number of fasted days associated with receiving prenatal consultation and the coefficients in Panel B reflect the estimated change in probability to begin fasting associated with receiving prenatal consultation. The table’s columns display results from three log-normal hurdle regressions that differ in their set of included covariates. The preferred specification in column (3) controls for all covariates. Controlling for more covariates takes better care of self-selection, i.e., the fact that women who are inclined to fast are more likely to ask for a consultation on this. Panel A shows that among women who fasted at least one day during pregnancy, prenatal consultation on Ramadan was associated with an average reduction in the number of fasted days by 10.8 days. With an average of 16.9 fasted days among fasting women (see Table 1), this implies a reduction in the number of fasted days by 64 %. The results were stable to the robustness checks additionally adjusting for fasting during past pregnancies and information seeking behavior (table A.6).

**Table 5:** Prenatal consultations on Ramadan fasting and fasting behavior of pregnant women: Results from LH regressions.

	(1)	(2)	(3)
*Panel A: Estimated fasting days among women who fasted during pregnancy (number of fasted days)*			
Prenatal consultation	-6.724 (4.069)	-9.293* (4.108)	-10.798** (4.202)

*Panel B: Estimated fasting probability (fasted at least one day)*			
Prenatal consultation	0.208** (0.057)	0.171** (0.055)	0.096 (0.050)
Socio-demographic control variables	No	Yes	Yes
Pregnancy, household, and religiosity controls	No	No	Yes

Notes: Each column presents results from a separate two-stage LH regression. Panel A presents results from the second stage and panel B results from the first stage. 321 observations contribute to the panel B results and 119 observations to the panel A results. Estimates are presented as average partial effects. Standard errors are in parentheses. Socio-demographic controls include education, employment type, age, age squared, first-generation status. Pregnancy, household, and religiosity controls include nulliparity, trimester overlap, daily veiling, partner’s objection, and other household members fasting.

* p<0.05; ** p <0.01

Regarding the binary fasting decision, the uncontrolled regression (Panel B, column (1)) suggests a high degree of self-selection: women receiving a prenatal consultation on Ramadan were 20.8 percentage points more likely to have fasted. This is unlikely to reflect an effect of the prenatal consultation but rather fits with the interpretation that women with the intention to engage in Ramadan fasting during pregnancy are also more likely to discuss fasting with a prenatal caregiver. This estimate is reduced in size and loses in significance upon the inclusion of covariates (Panel B, columns (2) and (3)).

The binary fasting decision is much more sensitive to the selection into treatment than the continuous measure. This is because consultations may have taken place while Ramadan was already ongoing. Consider a woman who stopped fasting after the issue was discussed with her gynecologist. This woman will have reported to have done some fasting, yet the prenatal consultation resulted in a lower number of fasting days. This is not picked up in the binary fasting decision variable, so we only observe a reduction in days fasted, but not in the probability of having fasted at least one day. Furthermore, having a more concrete reason, women who already had started fasting could be more likely to then consult their practitioner. However, the decision to discuss with a healthcare professional probably does not strongly depend on the number of days that a woman plans to fast or has already fasted.

Put together, this suggests that no clear conclusion on the effectiveness of consultations on the binary fasting decision can be drawn. What can be concluded, however, is that prenatal consultations did reduce the number of days that women fasted. The reported reduction by 10.8 days is likely an underestimate of the true effect since some self-selection remains regarding which women did vs did not receive consultations on Ramadan – even after including a set of covariates.

We more formally investigate potential bias from self-selection using Oster’s method, to assess to what extent adding covariates changes the coefficient of interest. Between the uncontrolled and the controlled regressions (cf. columns (1) and (3)), the coefficient changed. The question is what would have hypothetically happened to the coefficient if even more relevant covariates could have been added. The results of the Oster test statistic show that such (unobservable) residual confounders would not only have to be almost six times as important as the included covariates in explaining the outcome variable, but they would have to work in the opposite direction from the included covariates to nullify the detected association with the number of fasted days (online appendix table A.5). Furthermore, the Oster test results confirm our suspicion that the estimated positive association with the probability of beginning fasting cannot be interpreted reliably.

## 5 Discussion

This study examines the Ramadan fasting behavior during pregnancy among first- and second-generation Muslim immigrants in Germany. Among a sample of 326 women who had been pregnant during a Ramadan, 36.5 % had fasted. The average woman who fasted did so for 17 days.

Our documenting of the fasting decisions that women made, as well as the reasons behind these, add to the evidence on Ramadan fasting among pregnant Muslims in Europe. Fasting rates did not differ by educational level, age or parity, but they were lower among full-time employed women, perhaps due to challenges in combining a working life with fasting while pregnant. Fasting rates were also considerably higher among women for whom Ramadan occurred during their first pregnancy trimester. While previous studies mostly interviewed pregnant Muslims with an overlap between Ramadan and pregnancy in the second or third pregnancy trimester (Petherick et al., 2014, Savitri et al., 2014, Savitri et al., 2018), our findings suggest that it is important to ensure that future studies include information on all pregnancy stages. This is particularly relevant since health effects in response to Ramadan during pregnancy are often concentrated among those exposed in the first pregnancy trimester, so that understanding decision-making processes and determinants of fasting for this group can yield important insights for the design of migration sensitive prenatal care settings (Pradella et al., 2024). Our descriptive findings suggest that generally healthier women are more likely to select themselves into Ramadan fasting during pregnancy compared to women that had current conditions, ailments or were physically affected by the current pregnancy (see Table 2). This pattern suggests that previous findings on associations between maternal Ramadan fasting and health outcomes at birth or later in life may be biased towards zero in studies that rely on self-reported fasting behavior, particularly when controlling only for a limited set of confounders that influence both the maternal decision to fast and offspring health outcomes.

Although fasting is less common among second-generation immigrants, indicating a potential decline in fasting rates over time, the considerably higher fasting rates observed among the recent wave of predominantly Syrian immigrants suggest an opposing trend. In general, our findings are in line with previous studies on Ramadan during pregnancy in Europe documenting that fasting rates can differ greatly by country of origin. This observation was also made in the Netherlands (Savitri et al., 2014) and the UK (Petherick et al., 2014). While this observation is not surprising, since customs and traditions vary considerably across the globe, it has implications for the transfer of our results. Even though the fasting rate in our sample is similar to those found in the Netherlands (54 %) and the UK (43 %), drawing conclusions about fasting rates in other countries in the past or future has to be done with caution. For example, the largest share of Muslims in the UK is of Bangladeshi or Pakistani descent, as in Petherick et al. (2014).

While several previous studies reported on fasting rates among pregnant Muslims, this study went beyond a documentation of health behaviors among pregnant Muslims by exploring the underlying reasons as well as, for the first time, the role of prenatal care in forming decisions on maternal fasting during pregnancy. In any decision regarding health-related behaviors, besides an individual’s own attitudes, her subjective norms, i.e., her beliefs about what relevant other people may think about some behavior, play a role (McEachan et al., 2016). Whether a behavioral intention that results from attitudes and subjective norms is enacted depends on potential environmental constraints and behavioral control (Fishbein and Ajzen, 2011). The idea that fasting is mandatory during pregnancy appears to be no such constraint. This does not align with most Islamic religious interpretations according to which pregnant women are exempt if they have concerns about their own or their baby’s health. While many women cited religion as a reason for fasting, only very few say they are fasting because they have to.

Family and friends are listed as sources of information on Ramadan fasting during pregnancy among less than one-third of women. This is still much higher, though, than the share asking religious experts. The main source of information that was cited is the internet. In general, our findings suggest that pregnant Muslim women in Germany tend to make independent decisions regarding fasting during pregnancy, indicating a high level of perceived behavioral control over their choices. For instance, nearly half of those who fasted did so despite knowing that their partner objects to this.

All of this suggests that the opinions of relevant others (the subjective norms) are not the sole or main driver of women’s fasting decisions. Instead, the women’s own attitudes appear essential for these decisions. One underlying component of such attitudes are feelings, such as those stemming from religion or tradition. Another essential element are beliefs that can be based on correct or on incorrect information. Particularly relevant here are women’s beliefs about potential health effects. There appears to be a gap between these and research showing that Ramadan fasting during any phase of pregnancy can have long-run adverse effects on the health of the child (Pradella et al., 2024, Mahanani et al., 2021). Among fasting women, a majority expected there to be no, or even positive health effects on the child. Among women who did not fast, this picture was roughly flipped. The importance of these beliefs is corroborated by many women deciding on fasting based on how they felt physically. However, maternal fasting may be harmful to fetal development even if the woman feels no immediate repercussions to her physical well-being. The fact that many women reported beliefs that contradict scientific evidence on health effects of Ramadan during pregnancy underscores how important it is to provide accurate information.

Given that many women actively searched for information on fasting during pregnancy, but in most cases were unaware of potential adverse offspring health consequences, it seems sensible that prenatal caregivers should inform Muslim women on the potential effects of fasting during pregnancy. Since basically all pregnant women interact with healthcare professionals during their pregnancy and attend routine visits in Germany, we furthermore evaluated to which extent fasting decisions are amenable to advice given by prenatal caregivers. Most women did not consult with their prenatal care provider about Ramadan fasting (33.5 % in our sample did do so). Education, age and employment were not correlated with whether such consultations had taken place. This study did not involve a randomized trial regarding the effectiveness of consultations. This means that women could have self-selected into the treatment group (i.e., prenatal consultations). Once this is statistically taken into account, our results showed that discussing fasting with a healthcare professional is associated with a reduction in the number of days fasted by about 10.8 fewer days. This implies that prenatal caregivers can indeed serve as an important source of information that affects women’s fasting decisions. This should ideally occur before they become pregnant, as many women cited not yet knowing about being pregnant as the reason why they fasted. Also, early pregnancy has been shown to be the most sensitive phase for various prenatal exposures, including Ramadan.

This study opens important avenues for future research in immigration settings. While we measured actual fasting behavior and reasons for or against fasting, a more comprehensive overview of how women take their fasting decisions necessitates a more thorough investigation of how attitudes are formed, which relevant others play what role in the final decision, and what environmental constraints and behavioral control parameters are relevant. Given the study setting (interviewing women directly around delivery), this was not feasible and is left to future research. Moreover, while our results suggest that it is sensible to add Ramadan and other forms of intermittent fasting to the list of topics that should be addressed by medical practitioners according to maternity care guidelines, this also requires a sensitization of prenatal caregivers to e.g. the timing of Ramadan. Currently, it is unclear how well-informed prenatal caregivers in Germany (or other Western countries) are about Ramadan and associated customs. Given our findings that prenatal advice is associated with fasting during pregnancy, future research should aim to develop targeted interventions in randomized controlled trials - ideally while preserving the authenticity of patient-caregiver communication.

More research on successful patient-caregiver interactions appears to be pivotal. Exploring the patient’s personal situation and providing evidence in an open-ended, cooperative discussion is the essence of shared decision-making (SDM), which is a framework for patient-caregiver interactions in settings of relative uncertainty (Politi et al., 2013, Charles et al., 1999) that has gained increasing attention in the literature in recent years (Bomhof-Roordink et al., 2019). SDM has been proposed as a framework for patient-prenatal caregiver interactions in the context of Ramadan fasting (Shahawy et al., 2023). Discussing dietary choices during pregnancy while carefully exploring a sensitive issue and providing evidence to the patient is in line with the approach of SDM. The costs of a systematic implementation in routine prenatal care need to be evaluated in relation to resulting maternal behaviors and intentions.

This study has some limitations. First, we did not instruct medical practitioners about the content of the advice that they gave. They hence followed any approach that they deemed best. Similarly, we did not randomize pregnant women into prenatal consultation. This meant that we could not study the effects of giving advice in an optimized setting. We controlled for a rich set of covariates and our findings are robust to sensitivity analysis using the Oster method. Furthermore, the stepwise inclusion of socio-demographic control variables induces a pattern suggesting that some bias from self-selection is mitigated. Nevertheless, the possibility for residual confounding cannot be fully ruled out in the absence of randomized assignment of prenatal consultation.

At the same time, an advantage of this approach is that we analyze real-world evidence on “natural” patient-caregiver interactions, rather than a pre-manipulated intervention (Luce et al., 2010). Everyday patient-physician interactions under normal and unaltered conditions may differ from those in a controlled experiment (Barnish and Turner, 2017). Moreover, none of the advice given by prenatal caregivers was encouraging of fasting during Ramadan. Given our findings that prenatal advice is associated with fasting during pregnancy, future research should aim to develop targeted interventions in randomized controlled trials - ideally while preserving the authenticity of patient-caregiver communication. To assess whether the intervention altered beliefs about maternal and offspring health effects, the experiment should carefully assess pre- and post-intervention beliefs.

Second, as fasting was self-reported, reporting errors due to social desirability bias could occur. Krumpal (Krumpal, 2013) shows that the circumstances during the interview and how the interviewer is perceived largely govern the extent to which social desirability bias may occur. The interview team was comprised of both veiled and unveiled women. Comparing the fasting rates among veiled and unveiled interviewers showed no significant difference in fasting rates. This suggests that justification bias – that women may feel compelled to not answer the fasting questions truthfully to conform to what they believe the interviewer wants to hear – was unlikely to be a problem.

To conclude, many factors influence a woman’s fasting decision during pregnancy. Prenatal consultation appears to update the patients’ knowledge on Ramadan during pregnancy, translating into changes in the underlying decision-making process and subsequently changed fasting behavior during pregnancy. Addressing Ramadan during prenatal care can thus help women make informed choices. Therefore, healthcare providers to families of reproductive age can be advised to inform themselves about the upcoming Ramadan dates and actively talk to their Muslim patients about Ramadan fasting during pregnancy.

## Funding

The survey study was supported by the German Research Foundation [DFG, grant number 260639091 awarded to Reyn van Ewijk]. The funders had no role in study design, data collection and analysis, interpretation of data, decision to publish, or preparation of the manuscript.

## Data availability

This study draws on a relatively small sample, and the dataset contains combinations of variables that could potentially lead to the identification of individual participants. To construct key variables central to our analysis such as maternal age at childbirth, we required sensitive information, including the exact birth dates of both mothers and their children. Given the low number of daily births to Muslim parents in the study setting and the possibility of identifying Muslim participants by surname, public data sharing would put participant confidentiality at risk. Researchers who meet the requirements for accessing sensitive data may request access through the Ethics Committee of Johannes Gutenberg University Mainz, Department of Business and Economics.

## CRediT authorship contribution statement

**Paul Witte:** Writing – review & editing, Writing – original draft, Visualization, Methodology, Formal analysis, Data curation. **Fabienne Pradella:** Writing – review & editing, Writing – original draft, Validation, Project administration, Methodology, Investigation, Data curation, Conceptualization. **Reyn van Ewijk:** Writing – review & editing, Writing – original draft, Validation, Supervision, Project administration, Investigation, Funding acquisition, Conceptualization.

## Declaration of competing interest

The authors declare that they have no known competing financial interests or personal relationships that could have appeared to influence the work reported in this paper.

## Appendix Supplementary materials

Image, application 1
