# Supplementary material for: The impact of healthcare provision on immigrant pregnancy behaviors: the case of Ramadan fasting in Germany
Source: J Migr Health. 2025 Jul 25;12:100349. doi: 10.1016/j.jmh.2025.100349 (PMC12336805; doi:10.1016/j.jmh.2025.100349)
Supplement: Supplementary file 1 [file mmc1.docx]

**Online appendix**

**A.1 Regression model**

We consider three models that can be applied to corner solution problems. First, the type-1 Tobit model is a natural candidate (Tobin 1958), but does not allow the estimation of differential effects on the extensive (i.e. whether a woman fasted at all during pregnancy) and the intensive margin (i.e. how many days a woman fasted), while the disentanglement of these is central to our research.

Second, the zero-truncated-normal hurdle model (TNH) as proposed by (Cragg 1971) estimates non-zero participation, i.e. the probability to fast at least one day during pregnancy by a probit regression. The number of days fasted (conditional on having cleared the zero-hurdle of having fasted at least one day) is estimated by a truncated normal regression:

| $P\left( y_{i}>0 \right\vert{A_{i},x}_{i})=\Phi(\alpha_{1}A_{i}+\alpha_{2}x_{i})$ | (1) |
| --- | --- |
| $E\left( y_{i} \right\vert y_{i}>0,{A_{i},x}_{i})=\left( \gamma_{1}A_{i}+\gamma_{2}x_{i} \right)+\sigma\lambda\left( \frac{\gamma_{1}A_{i}+\gamma_{2}x_{i}}{\sigma} \right)$ | (2) |

Here, $y_{i}$ describes the number of fasted days during pregnancy, $A_{i}$ is a binary indicator for whether a prenatal consultation took place and $x_{i}$ contains the set of observable covariates. $\Phi$ is the standard normal cumulative distribution function transforming the respective index function, $\sigma$ contains the estimated standard error of the regression and $\lambda$ is the inverse Mills ratio. Parameter estimates are obtained by maximum-likelihood estimation (MLE).

From the TNH, two distinct quantities are obtained. First, the associations between prenatal consultation on Ramadan and fasting at least one day estimated by a probit regression model (equation (1)), which we will refer to as the extensive margin. Second, the effects of prenatal consultation on the number of days fasted conditional on whether a woman fasted at least one day estimated by a truncated normal regression model (equation (2)), which we will refer to as the intensive margin. The truncation point is set at zero.

The third model we consider is the log-normal hurdle (LH) model, which estimates the natural logarithm of the outcome via OLS for the non-truncated (greater than zero) responses (Cragg 1971).

| $E\left( y_{i} \right\vert y_{i}>0,{A_{i},x}_{i})=exp\left( \gamma_{1}A_{i}+\gamma_{2}x_{i} \right)$ | (3) |
| --- | --- |

In the LH model, the binary participation is still estimated via a probit model as in the TNH.

**A.2 Detailing the Oster (2019) method**

The test statistic compares the raw consultation-fasted days association with the adjusted association from the regression analysis. Considering how the coefficient of interest, as well as the explained variance ($R^{2}$) change, the test statistic indicates how important unobserved residual confounders would have to be in order to explain away the reported effect to the point where the null hypothesis (no effect of prenatal consultation on number of days fasted) would no longer be rejected.

To tie the coefficient movements upon the inclusion of observables to the respective regression’s $R^{2}$ metric, possible maximum values for $R^{2}$ are to be specified. Oster (2019), based on analyzing data from previous studies, proposes an $R_{max}$of 1.3 times the $R^{2}$ metric of the respective controlled regression.

Oster thus formulates approximation (4),

| $\beta^{*}\approx\tilde{\beta}-\delta[\beta-\tilde{\beta}]\times\frac{R_{max}-\tilde{R}}{\tilde{R}-R}$ | (4) |
| --- | --- |

where $\beta^{*}$ is the true effect, $\beta$ is the univariate effect estimate, $\tilde{\beta}$ is the controlled effect estimate, $R$ is the univariate model’s share of explained variance, $\tilde{R}$ is the controlled model’s share of explained variance, and $\delta$ is the relevance of unobservable confounders relative to the included observables that would be required to obtain the true effect $\beta^{*}$. $R_{max}$ must be specified by the researcher and may in principle range from zero to one. This test can be used in two ways. First, by calculating the true effect $\beta^{*}$ when assuming values for $\delta$ (usually $\delta=1$, indicating that unobserved covariates are equally important as observed covariates). Second, obtaining $\delta$ when assuming the true effect $\beta^{*}$was zero, i.e., how relevant unobservable confounders would need to be in order for the true effect to be zero.

The test statistic requires output from linear ordinary-least squares (OLS) regressions (Oster 2019). Regarding the regression models we consider, replicating the two stages of the TNH (estimated by MLE) with linear regression might be problematic if model misspecification results in biased and inconsistent estimates. For the first stage estimated by a probit regression model, the replication with OLS yielding a linear probability model (LPM) is less of a concern. LPMs are frequently used in the literature, mostly due to ease of interpretation of the marginal effects. They do have well-known limitations such as the assumption of constant marginal effects or the potential for predicted probabilities outside the zero-one interval, but marginal effect estimates (averaged or at the mean) are usually close to the ones obtained by probit or logit models (Horrace and Oaxaca 2006).

For an OLS replication of the truncated normal regression at the second stage of the TNH, consider a general version of equation (2):

| $E\left( y_{i} \right\vert y_{i}>0,x_{i})=x_{i}^{'}\beta+\sigma\lambda\left( \frac{x_{i}^{'}\beta}{\sigma} \right)$ | (5) |
| --- | --- |

An OLS replication as in

| $E\left( y_{i} \right\vert y_{i}>0,x_{i})=x_{i}^{'}\beta$ | (6) |
| --- | --- |

is mis-specified in this case because it omits the truncation correction $\sigma\lambda\left( \frac{x_{i}^{'}\beta}{\sigma} \right)$. This renders the LH model more attractive for applying the Oster method, as the second stage is estimated by OLS.

**A.3 Country of birth stratification**

| **Table A.3.** Ramadan fasting behavior by country of birth | | | | |
| --- | --- | --- | --- | --- |
|  | | | | |
| Country of birth | Fasting at least one day (Percent) | Number of days fasted  (Mean) | Fasted days if fasted at least one day  (Mean) | Observations |
| Germany | 16.5 | 2.1 | 12.6 | 91 |
| Syria | 61.5 | 11.5 | 18.6 | 52 |
| Morocco | 66.7 | 12.2 | 18.9 | 48 |
| Turkey | 20.5 | 2.8 | 13.4 | 39 |
| Other Arabic countries | 34.8 | 5.8 | 16.6 | 46 |
| South or Central Asia | 29.6 | 4.8 | 16.1 | 27 |
| Other countries | 34.8 | 6.6 | 19 | 23 |
| Total | 36.7 | 6.2 | 16.9 | 326 |
|  | | | | |

**A.4 Information seeking stratification**

| **Table A.4.** Information seeking: fasting behavior and health beliefs among fasting women | | | | | | | |
| --- | --- | --- | --- | --- | --- | --- | --- |
|  | | | | | | | |
|  | | | (1) Consultation only | (2) Information only | (3) Both | (4) Neither | (5) P-value for between-group differences |
|  |  |  |  |  |  |  |  |
| **Avg. fasting days** | | | 16.79 | 17.33 | 14.67 | 19.39 | 0.286 ^b^ |
| **Beliefs about maternal health effects** | | |  |  |  |  |  |
| Positive | | | 0.07 | 0.18 | 0.09 | 0.08 |  |
| No effect | | | 0.43 | 0.33 | 0.42 | 0.54 | 0.788 ^a^ |
| Negative | | | 0.21 | 0.26 | 0.24 | 0.24 |  |
| **Beliefs about offspring health effects** | | |  |  |  |  |  |
| Positive | | | 0.07 | 0.22 | 0.24 | 0.11 |  |
| No effect | | | 0.57 | 0.33 | 0.49 | 0.46 | 0.578 ^a^ |
| Negative | | | 0.14 | 0.15 | 0.19 | 0.24 |  |
| Observations | | | 14 | 27 | 41 | 37 |  |
| Notes: P-value based on a: $\chi^{2} -$test, b: ANOVA. | | | | | | | |

**A.5 Oster test statistic results**

| **Table A.5**. Applying the Oster method to OLS replications of the log-normal hurdle model | | | | | | |
| --- | --- | --- | --- | --- | --- | --- |
|  | | | | | | |
|  | | | | | | |
| Treatment variable | (1)  Univariate effect | (2)  Controlled effect | (3)  99.5% CI  (of (2)) | (4)  Bias-adjusted β for δ = 1 | (5)  Bias-adjusted β for δ = - 1 | (6)  δ for β = 0 |
| *Panel A: OLS results for log(number of fasting days)* | | | | | | |
| Prenatal  consultation  $R_{max}$= 0.374 | -0.333 | -0.581** | [-1.097;  -0.064] | -0.683 | -0.487 | -5.769 |
| *Panel B: Linear probability for binary fasting decision* | | | | | | |
| Prenatal  consultation | 0.208** | 0.102 | [-0.057; 0.260] |  |  |  |
| $R_{max}$= 0.419 |  |  |  | 0.062 | 0.139 | 2.341 |
| Notes: The specifications denoted as controlled include all control variables from the truncated normal hurdle model (main specification).  * p<0.05; ** p <0.01 | | | | | | |
|  | | | | | | |

**A.6 Results of robustness checks including additional covariates**

| **Table A.6. Robustness checks** with additional control variables (having fasted during previous pregnancies (2) and general information seeking (3)) | | | |
| --- | --- | --- | --- |
|  | | |  |
|  | (1) Main specification | (2) Additional control variable (1) | (3) Additional control variable (2) |
| *Panel A: Estimated fasting days among women who fasted during pregnancy (number of fasted days)* | | |  |
| Prenatal consultation | -10.798** (4.202) | -15.210** (4.679) | -8.685** (3.675) |
| *Panel B: Estimated fasting probability (fasted at least one day)* | | |  |
| Prenatal consultation | 0.096  (0.050) | 0.079  (0.059) | 0.078 (0.052) |
| Socio-demographic control variables | Yes | Yes | Yes |
| Pregnancy, household, and religiosity controls | Yes | Yes | Yes |
| Fasting in previous pregnancies | No | Yes | No |
| Independent information seeking | No | No | Yes |
| Observations Panel A  Observations Panel B | 119  321 | 72  191 | 119  321 |
| Notes: ** p < 0.01, * p < 0.05. | | |  |
|  | | |  |

**References**

Cragg, John G. 1971. "Some statistical models for limited dependent variables with application to the demand for durable goods." *Econometrica: journal of the Econometric Society*:829-844.

Horrace, William C, and Ronald L Oaxaca. 2006. "Results on the bias and inconsistency of ordinary least squares for the linear probability model." *Economics letters* 90 (3):321-327.

Oster, Emily. 2019. "Unobservable selection and coefficient stability: Theory and evidence." *Journal of Business & Economic Statistics* 37 (2):187-204.

Tobin, James. 1958. "Estimation of relationships for limited dependent variables." *Econometrica: journal of the Econometric Society*:24-36.
